# A systematic review and meta-analysis of compassion fatigue among healthcare professionals before and during COVID-19 in Sub-Saharan Africa

**DOI:** 10.1371/journal.pgph.0003388

**Published:** 2024-06-21

**Authors:** Amir Kabunga, Eustes Kigongo, Raymond Tumwesigye, Samson Udho, Marvin Musinguzi, Walter Acup, Anna Grace Auma, Anne Ruth Akello, Ponsiano Okalo, Jannat Nabaziwa, Enos Mwirotsi Shikanga

**Affiliations:** 1 Department of Psychiatry, Lira University, Lira City, Uganda; 2 Department of Environmental Health and Disease Control, Lira University, Lira City, Uganda; 3 Department of Nursing, Lira University, Lira City, Uganda; 4 Department of Community Health, Lira University, Lira City, Uganda; 5 Department of Education Psychology, Moi University, Eldoret City, Kenya; Fatima College of Health Sciences, UNITED ARAB EMIRATES

## Abstract

Compassion fatigue is a significant concern globally, particularly in Sub-Saharan Africa, where the COVID-19 pandemic exacerbated existing challenges, placing unprecedented strain on healthcare professionals. This study systematically estimated the prevalence of compassion fatigue among healthcare professionals before and during COVID-19 in Sub-Saharan Africa. A systematic review was conducted using keywords in PubMed, ScienceDirect, Google Scholar, and grey literature, covering all literature published between 2012 and December 30, 2023. The search team independently conducted study selection, quality assessments, data extractions, and analysis of all included studies. The systematic review, reported following PRISMA guidelines, included 11 studies. The results show that the pooled overall prevalence of compassion fatigue in Sub-Saharan Africa was 70% (95% CI: 57–82, I^2^ = 88.37%). The highest prevalence was found in Eastern Africa at 74% (95% CI: 55–93, I^2^ = 94.40%), compared to 64% in Southern Africa (95% CI: 49–79, I^2^ = 59.01%). Nurses reported the highest rates of compassion fatigue at 80% (95% CI: 57–100, I^2^ = 34.77%), followed by general healthcare professionals at 59% (95% CI: 22–97, I^2^ = 94.11%) and nursing students at 50% (95% CI: 35–64, I^2^ = 0.00%). Before COVID-19, the overall prevalence of compassion fatigue was 66% (95% CI: 41–91, I^2^ = 27%). During COVID-19, this increased to 74% (95% CI: 63–85, I^2^ = 88.73%). Our results indicate that nearly 3 in 4 healthcare professionals in Sub-Saharan Africa experience compassion fatigue, and this prevalence increased due to the pandemic. The high prevalence underscores the importance of addressing and mitigating compassion fatigue to support the mental health and emotional well-being of healthcare professionals dedicated to helping others in challenging circumstances.

**Systematic registration:** PROSPERO. REG No: CRD42023449462.

## Introduction

Compassion fatigue is a serious global challenge among healthcare professionals dealing with poor health outcome in hospital settings [1]. Before COVID-19, more than half of healthcare professionals in high income countries experience compassion fatigue [2] and there is growing evidence that it is higher in developing countries [3, 4]. The COVID-19 pandemic of 2019 caused unprecedented challenges for healthcare professionals and placed a great deal of strain on healthcare systems around the world. Sub-Saharan Africa is not an exception to this global problem [5]. Throughout the pandemic, healthcare professionals in Sub-Saharan Africa led the way in combating COVID-19, continuously providing care to patients in spite of scarce resources, a heavy caseload, and personal risk [5]. The extreme burden this essential responsibility placed on healthcare professionals—a strain that extended beyond the physical demands of their jobs—led to the emergence of compassion fatigue.

Compassion fatigue also called secondary traumatic stress disorder or vicarious trauma is the phenomenon of depletion and dysfunction in healthcare professionals brought on by extended exposure to work-related stress and direct contact with traumatized patients and families [6, 7]. Most medical definitions of compassion fatigue focus on the loss of a healthcare professional’s ability to interact compassionately with patients [8]. According to the widely accepted conceptual model, professional quality of life (ProQOL), which is characterized as having both positive (compassion satisfaction) and negative (secondary traumatic stress and burnout) elements, can be used to measure compassion fatigue. It usually happens after the healthcare professionals has invested a lot of time and effort (as they did during COVID-19) into caring for the patients under their care. Compassion fatigue is consistent with the diminished capacity for compassion brought on by excessive exposure to patients’ pain [9]. Compassion fatigue is an important issue because it can have an impact on an individual’s overall well-being, job satisfaction, and, eventually, the quality of patient care [10].

Healthcare professionals were exposed to unprecedented traumatic materials during the pandemic which could have worsened their quality of life aggravating pre-exiting problems such as compassion fatigue [11]. Compassion fatigue in medical professionals has typically been unnoticed, especially in developing nations, and the pandemic’s novelty creates a gap in the knowledge of the prevalence of compassion fatigue among healthcare professionals. Additionally, healthcare professionals in Sub-Saharan Africa are facing pressures that predate the arrival of COVID-19 [5]. These systems frequently struggle with low personnel, little resources, and a high incidence of infectious infections [12]. These difficulties were made more worse by the epidemic, which added new levels of stress, anxiety, and uncertainty [13]. As a result, the healthcare professionals in this area were subjected to a distinct combination of conditions that could have increased the likelihood of compassion fatigue and consequently compromised their capacity to provide high-quality healthcare services.

In Sub-Saharan Africa, where healthcare systems were already strained, the pandemic’s arrival further amplified existing issues, including inadequate personal protective equipment (PPE), increased patient volumes, and heightened emotional demands. Before the COVID-19 pandemic [13], studies in Sub-Saharan Africa had already highlighted concerning levels of emotional exhaustion among healthcare professionals [14]. However, the specific impact of compassion fatigue remained relatively understudied in this region. Understanding the prevalence and factors contributing to compassion fatigue among healthcare professionals is crucial for several reasons. First and foremost, healthcare providers experiencing compassion fatigue are at risk of reduced quality of care, decreased job satisfaction, and increased rates of turnover [15]. This not only impacts individual providers but also undermines the overall effectiveness and resilience of healthcare systems.

Although systematic reviews have been conducted on the impact of the COVID-19 pandemic on the mental health of healthcare professionals, few have examined dimensions of quality of life such as compassion fatigue in health professions [16], specifically in Sub-Saharan Africa. Limited information exists regarding the overall burden of compassion fatigue among healthcare professionals in this region before or during the COVID-19 pandemic. It remains unclear whether healthcare professionals were more susceptible to compassion fatigue before or during the pandemic. This systematic review and meta-analysis aims to present a pooled prevalence of compassion fatigue among healthcare professionals in Sub-Saharan Africa before and during the COVID-19 pandemic. Such an analysis would offer valuable insights into its prevalence and could inform the development of evidence-based interventions to prevent and manage compassion fatigue among healthcare professionals in the region.

## Methods

### Protocol

The protocol of study was registered on the International Prospective Registrar of Systematic Review (PROSPERO. REG No: CRD42023449462). The Preferred Reporting Items for Systematic Reviews and Meta-Analyses (PRISMA) guidelines was used for conducting this systematic review and meta-analysis [17].

### Search strategy

PubMed, ScienceDirect and Google scholar were the main databases searched (S1 Text). However, we also searched for gray literature in light of the study question. Additionally, the research team scanned reference lists for similar studies. To conduct a search between groups of words, the specific database thesaurus, for instance, medical and subject headings (MeSH), were used with a combination of keywords with the boolean operators “AND” and "OR.” These were searched for in the article title, abstract, and keywords. The search string was initially developed for the PubMed database and then modified for additional relevant databases (S1 Text).

### Eligibility criteria and study selection

The review considered observational studies reporting on the prevalence of compassion fatigue in Sub-Saharan Africa. We included: primary studies reporting on the prevalence of compassion fatigue in Sub-Saharan Africa; publications from 2012 to December 2023; all compassion fatigue scales; and articles published in English. The review excluded publications if they were editorials, letters to the editor, articles not related to the research topic (compassion fatigue), study protocols, or commentaries. All the searched articles and reports from the different sources were exported to Rayyan software for screening. The titles and abstracts of every article were evaluated by two authors following some preset inclusion criteria. All articles that were included at this stage were moved to full text screening, also done by two independent authors. In cases of disputes over whether to include or exclude an article by the two independent authors, the senior author made the final decision whether or not to include the study when the first two authors had disagreements. We created a flow process chart using the Preferred Reporting Items for Systematic Reviews and Meta-Analyses (PRISMA) guidelines to show the selection process of the review [18].

### Outcome measurement

The primary outcome was the prevalence of compassion fatigue. The second secondary outcome was comparisons of compassion fatigue scores before and during COVID-19 and regions among different categories of healthcare professionals.

### Data extraction

A pretested form was used to extract data from the included studies. The parameters reported included the study title, first author, publication year, sample size of the study, country and region, time of the study, study design, study participants, study data collection instrument, and findings, all of which were provided on this form. for each of the selected articles. Data extraction was conducted by two groups of authors who convened to discuss the results obtained to ensure correctness.

### Risk of bias assessment or quality of the study

Using an adapted Newcastle and Ottawa tool for the assessment of the quality of cross-sectional studies [19]. Based on previous studies, the tool has a good interrater agreement coefficient (ICC = 0.633, 95% CI: 0.387–0.802) [20]. The first author and second reviewer independently conducted a quality assessment of the included studies. This tool is appropriate for evaluating the quality of the articles included in this systematic review and analyzing seven areas, including the representativeness of the sample, sample size, response rate, screening tool, comparability, outcome assessment, and statistical test. Studies are rated out of a score of nine; any study above a score of seven is rated as a good quality study (S1 Table).

### Data synthesis and analysis

All data analysis was performed in Stata software version 17 (2021), StataCorp, LLC, College Station, Texas, USA. We examined whether it was possible to conduct a meta-analysis first by assessing clinical heterogeneity. We used the random effects model with a T2 calculator based on the residual maximum likelihood technique (REML). The pooled prevalence of compassion fatigue and corresponding confidence intervals from the original data were estimated and presented in a forest plot. where at least two studies reported on the sex, marital status, and level of education, their association was assessed with the level of compassion fatigue and reported as odds ratios and corresponding 95% confidence interval. Subgroup analysis was performed based on the potential causes of heterogeneity, including but not limited to region, COVID-19 period, and cadre of healthcare professionals. Potential publication bias and an assessment of small-study effects were assessed through visual inspection of funnel plot asymmetry and confirmed with the Egger’s test with p<0.05. Additionally, a leave-one-out sensitivity analysis was performed to assess the robustness of the results and identify studies that would distort the overall estimates so that they were removed.

## Results

**Fig 1:**
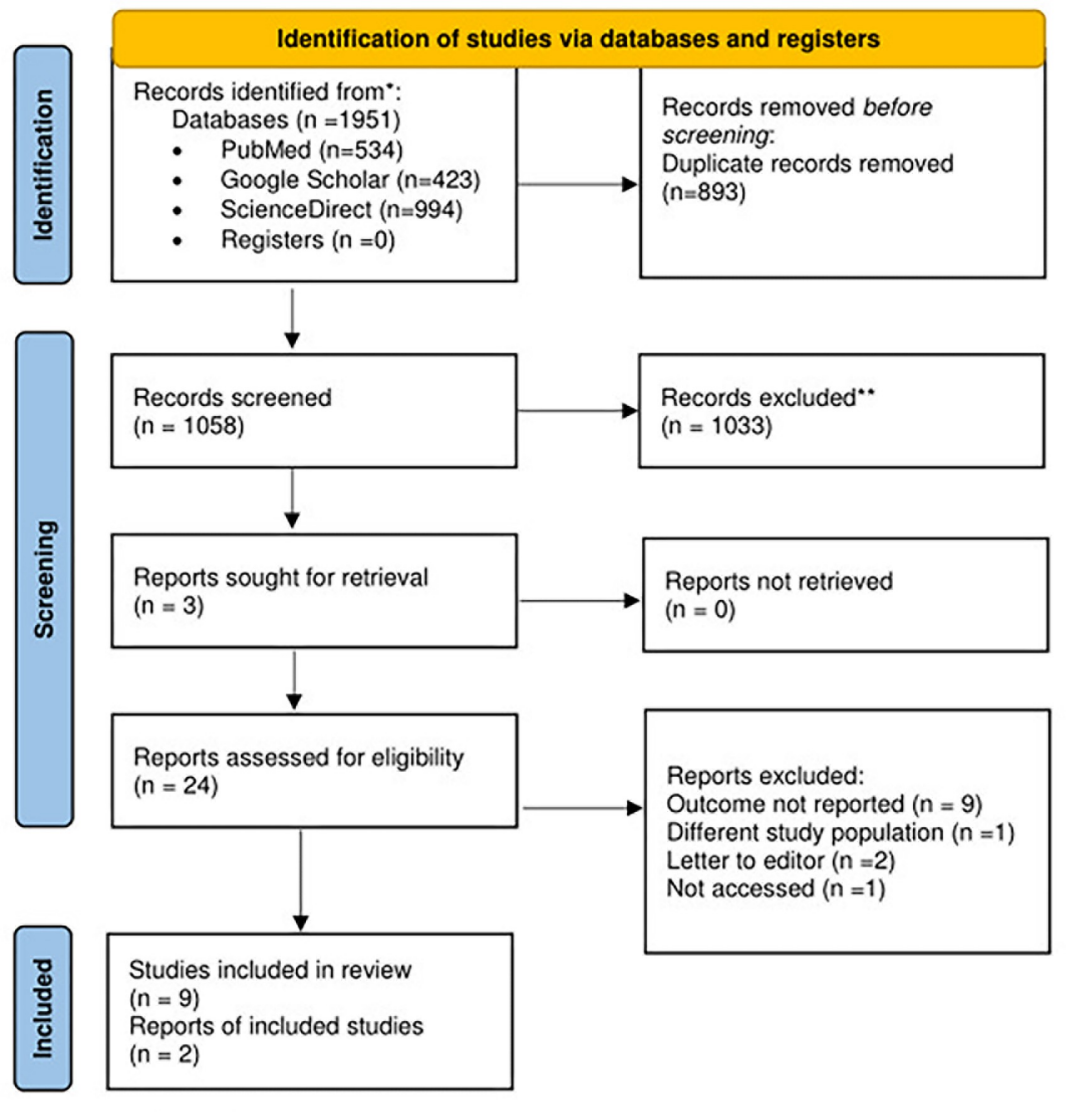
PRISMA flow diagram for included studies as of December 2023.

The systematic search yielded 1951 results. After removing the 893 duplicate files, 1951 articles remained. We screened the study abstracts and remained with 24 articles. Of the 24, 13 were excluded after full text screening because they did not report the main study outcome (9), were not among healthcare professionals (1), were letters to the editor (1), and were not accessed (1). The results are shown in Fig 1.

### Study characteristics

Table 1 shows that a total of 11 studies were included in the review. Studies were only from two regions: Eastern [3, 4, 21–24] and Southern [1, 25–28]. Out of the 11 studies, five were done before the COVID-19 pandemic [21, 24–26, 28] and six after the emergency of the pandemic [1, 3, 4, 22, 23, 27]. Most of the studies were conducted among nurses, while others were among general healthcare professionals and nursing students. The total sample size for the study was 2341 participants, ranging from 67 [26] to 410 [22]. Only two studies were assessed with poor quality [23, 28]. In the analyzed studies, the most significant methodological challenges were about representativeness of the study population, the sample size and the response rate.

**Table 1:** Characteristics of included studies.

Author	Year	Participants	Period	Country	Sampling	Gender	Sample size (n)	Prevalence	Tool	Quality
Donald [29]	2015	Healthcare workers	Pre COVID	Kenya	Simple random	251	345	100	CFST	Good
Teresa [25]	2016	Nurses	Pre COVID	South Africa	Simple random	81	83	71	ProQOL	Good
Mathias [26]	2017	Nursing students	Pre COVID	South Africa	Census	54	67	31	ProQOL	Good
Wentzel [28]	2017	Nurses	Pre COVID	South Africa	Stratified	81	83	65	ProQOL	Poor
Addisu [24]	2019	Healthcare professionals	Pre COVID	Ethiopia	Not reported	79	152	142	ProQOL	Good
Almaz [23]	2020	Nurses	Post COVID	Ethiopia	Purposeful	154	230	216	Not reported	Poor
Amir [3]	2021	Nurses	Post COVID	Uganda	Convenience	230	395	311	ProQOL	Good
Beatrice [27]	2021	Nursing students	Post COVID	South Africa	Not reported	102	108	56	ProQOL	Good
Mohammed [22]	2022	Nurses	Post COVID	Ethiopia	Simple random	216	410	298	ProQOL	Good
Amir [4]	2022	Nurses	Post COVID	Uganda	Simple random	230	395	311	ProQOL	Good
Phindile [1]	2023	Healthcare professionals	Post COVID	South Africa	Purposeful	59	73	41	ProQOL	Good

ProQOL = Professional quality of life, CFST = Compassion Fatigue/Satisfaction Self-Test

### Pooled prevalence of compassion fatigue

**Fig 2:**
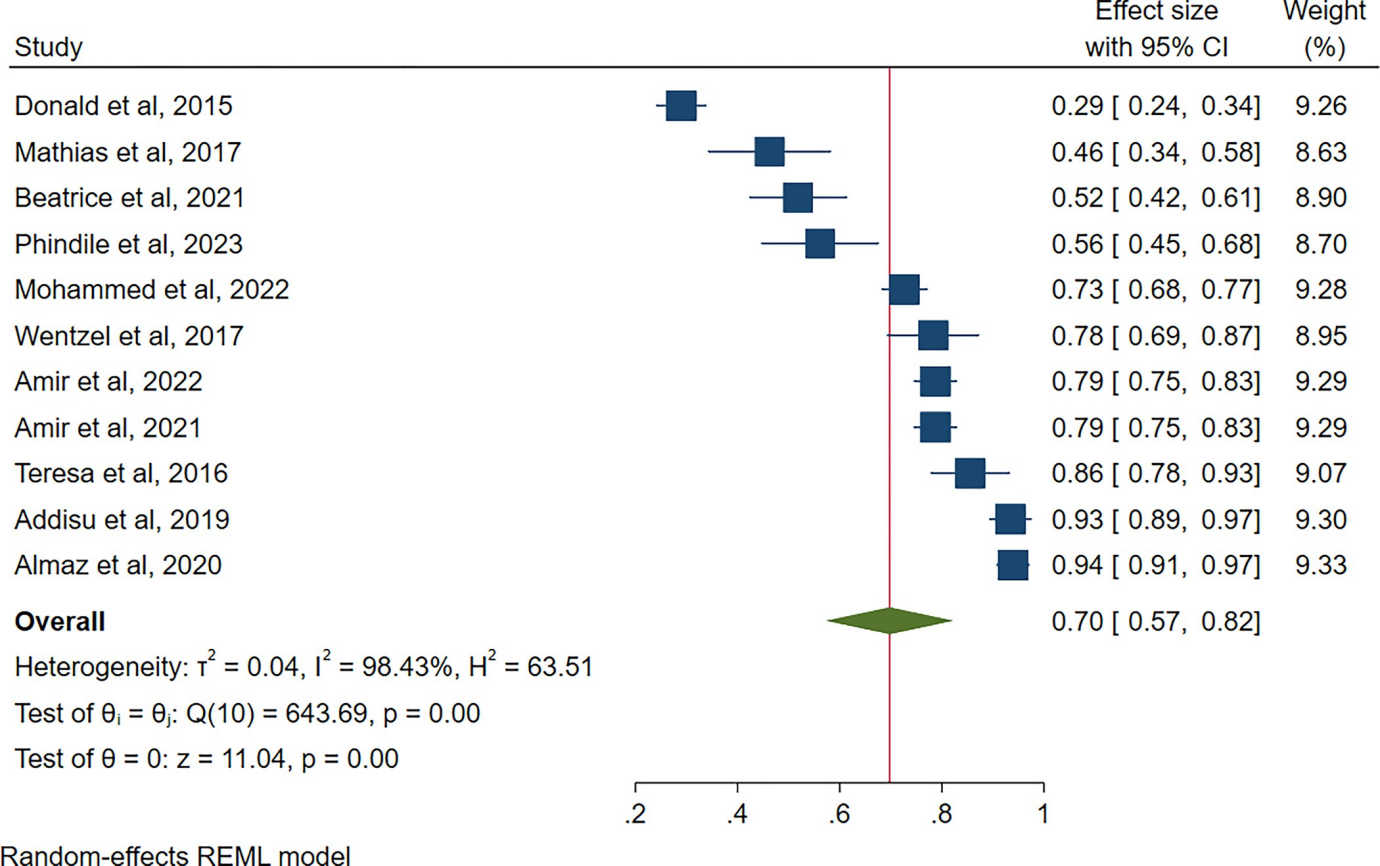
Pooled prevalence of compassion fatigue.

Fig 2 shows the overall prevalence of compassion fatigue in Sub-Saharan Africa was 70% (95% CI: 57–82, 1^2^ = 88.37%). From the included studies, the prevalence of compassion fatigue ranged from 29% [29] to 93% [24]. Compassion fatigue is measured based on scores that classify risk as low, average and high risk for compassion fatigue. This study combined average and high risk for the prevalence of compassion fatigue.

### Heterogeneity and Sub group

Heterogeneity was reported with an index (I^2^) of 88.73% and a p<0.001. Subgroup analyses were performed to assess the potential sources of the observed heterogeneity. The analysis for year of publication (before COVID-19 versus during COVID-19), study population, and geographical region was conducted.

### Prevalence of compassion fatigue in different geographical locations

**Fig 3:**
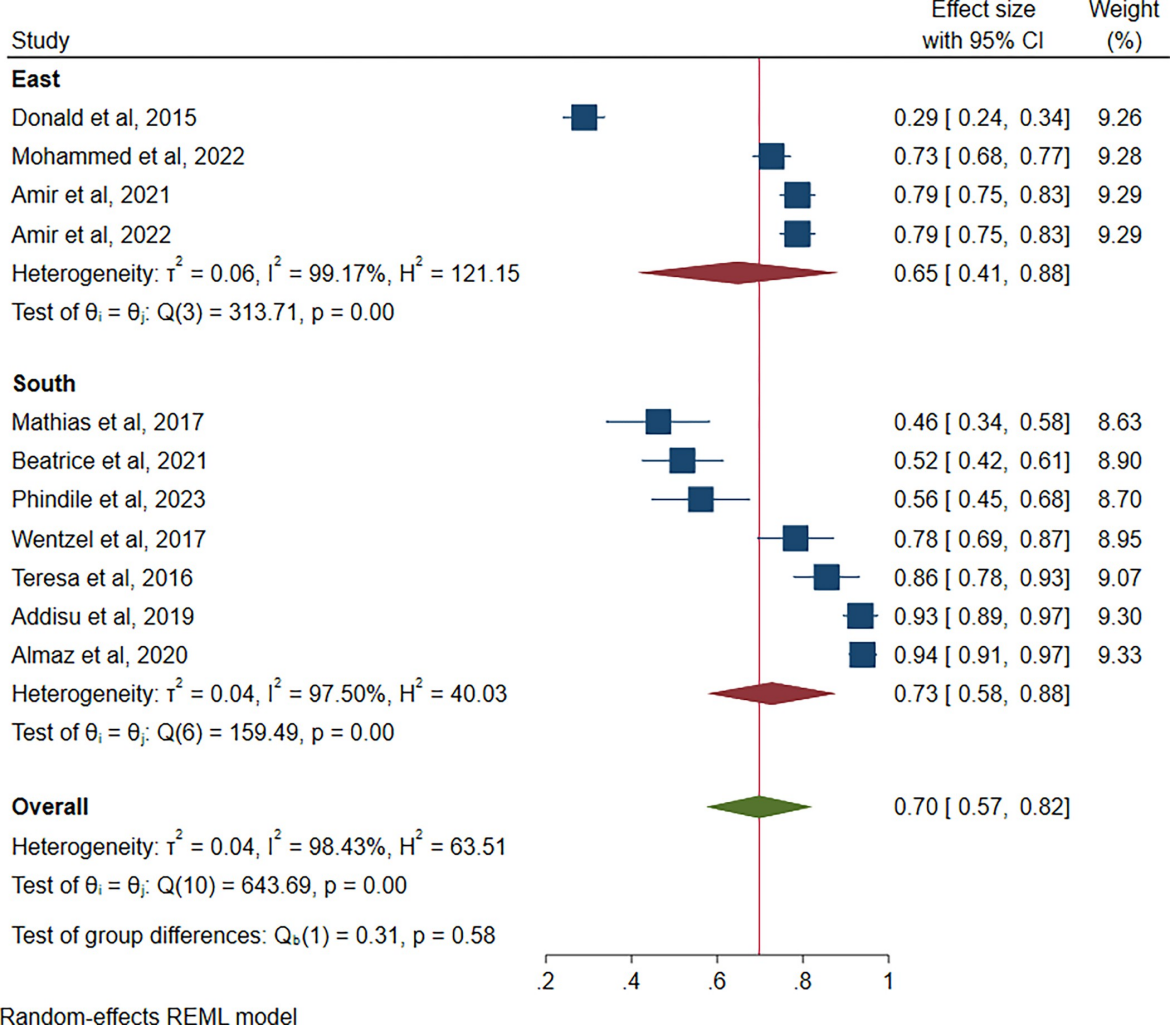
Prevalence of compassion fatigue by geographical location.

As shown in Fig 3, compassion fatigue among healthcare professionals was highest in eastern Africa at 65% (95% CI: 41, 88%, 1^2^ = 99.17%) as compared to 64% reported in southern Africa (95% CI: 58, 88%, 1^2^ = 97.50%).

### Prevalence of compassion fatigue among different categories of healthcare professionals

**Fig 4:**
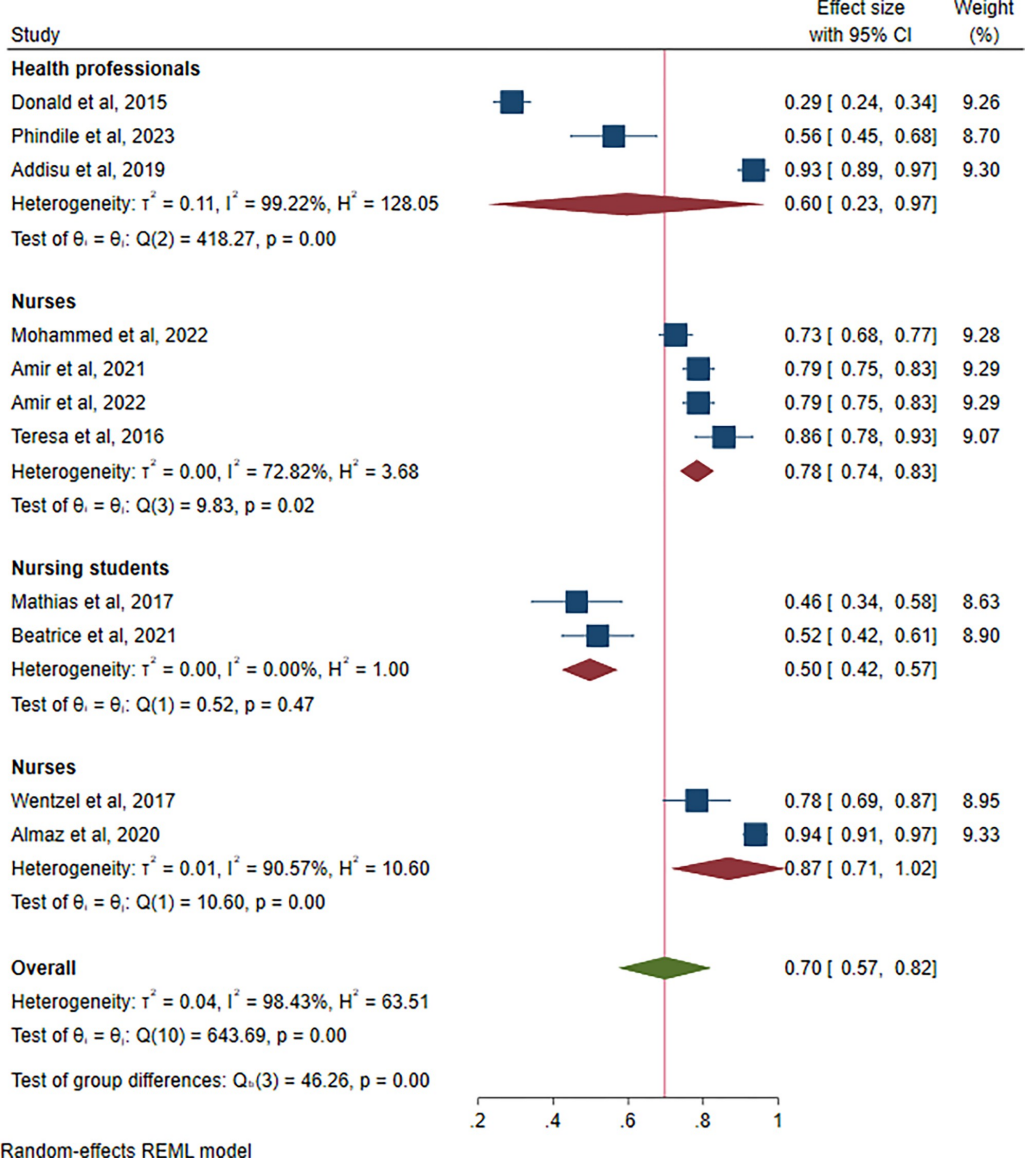
Prevalence of compassion fatigue by healthcare professionals.

Eleven studies compared compassion fatigue between general healthcare professionals and nurses. Among the 11, three studies reported compassion fatigue among general healthcare professionals, five reported compassion fatigue among nurses, and two reported compassion fatigue among nursing students. In comparison, nurses reported the highest rates of compassion fatigue at 87% (95% CI: 71, 100%, 1^2^ = 72.82%), followed by general healthcare professionals at 60% (95% CI: 23, 97%, 1^2^ = 99.22%), and lastly nursing students at 50% (95% CI: 42, 57%, 1^2^ = 0.00%), as shown in Fig 4.

### Prevalence of compassion fatigue before and during COVID-19

**Fig 5:**
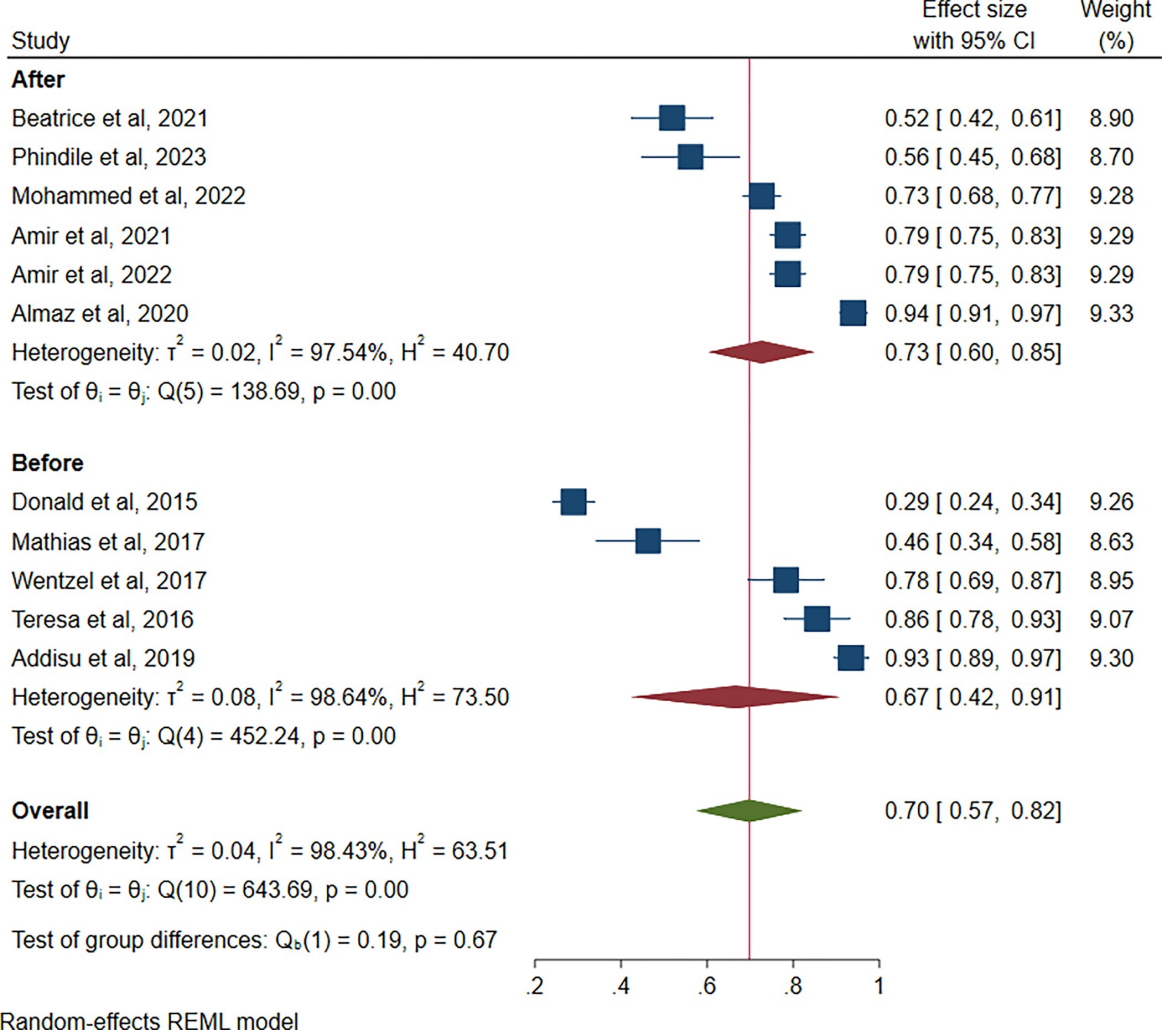
Prevalence of compassion fatigue before and during COVID-19.

Overall compassion fatigue prevalence before COVID-19 was found to be 67% (95% CI: 42, 91%, *1*^2^ = 98.64%) among healthcare professionals. Overall compassion fatigue during COVID-19 was shown to be prevalent in 73% (95% CI: 60, 85%, *1*^2^ = 97.54%) of the healthcare professionals, as shown in Fig 5.

### Sensitivity analysis

**Fig 6:**
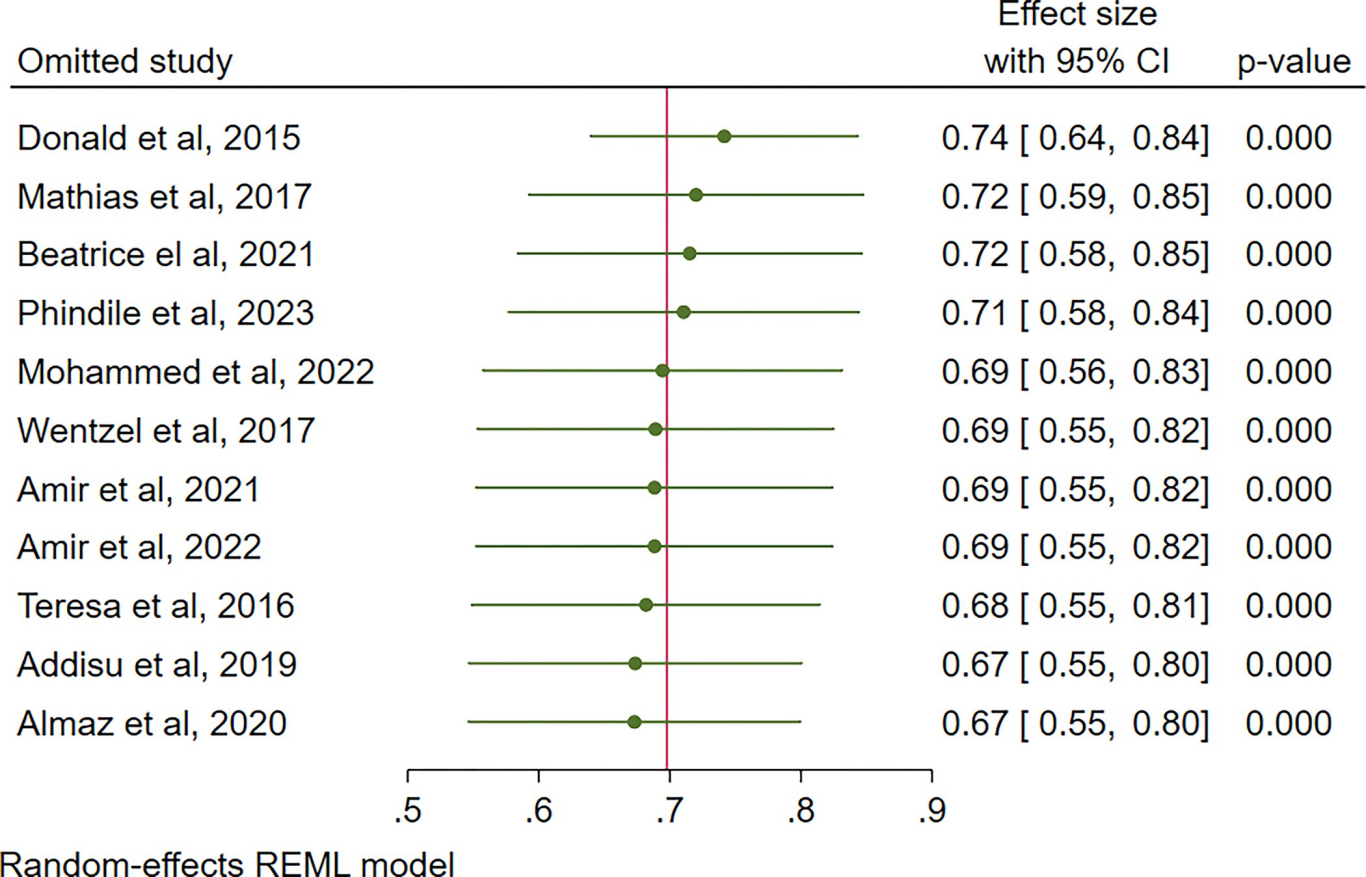
Sensitivity analysis.

The results of a leave-one-out sensitivity analysis were shown in Fig 6. This was done to re-estimate the effect of the remaining studies on the overall prevalence of compassion fatigue, after eliminating one study at a time. The results indicate that all 11 included studies had a prevalence ranging from 67% (95% CI: 54, 80%) to 75% (95% CI: 66, 85%), which is within the overall pooled estimate of 70% (95% CI: 57, 82%). Additionally, based on standard residuals, all the studies were within in -2 to +2 and no study was eliminated from the meta-analysis.

## Discussion

The findings of this systematic review and meta-analysis reveal a staggering prevalence of compassion fatigue among healthcare professionals in Sub-Saharan Africa, with an alarming 70% pooled prevalence, indicating the immense psychological toll exacerbated by the COVID-19 pandemic. This high prevalence suggests significant mental health challenges, impacting both well-being and care provision effectiveness. Comparing these findings to pre-pandemic levels, it’s evident that the strain on healthcare workers has intensified during the COVID-19 era. Notably, the study’s results show a higher prevalence of compassion fatigue compared to pre-pandemic rates. Accordingly, there is evidence showing that compassion fatigue among healthcare professionals worsens over time or has an upward trend [30]. The high risk of compassion fatigue in Sub-Saharan Africa among healthcare professionals is more striking, perhaps due to the COVID-19 pandemic. Our findings indicate a prevalence of compassion fatigue among healthcare professionals in Sub-Saharan Africa that surpasses rates observed in studies conducted prior to the COVID-19 pandemic [31]. Also, comparing these results with another relevant systematic review and meta-analysis [32], we find notable differences. The differences in prevalence rates could be attributed to the specific populations included in each study. These differences show the urgent need for targeted interventions to address and alleviate compassion fatigue among healthcare workers in Sub-Saharan Africa, especially in the wake of the COVID-19 pandemic, emphasizing the necessity of comprehensive support systems and resources to safeguard frontline workers’ well-being and ensure the provision of quality healthcare services to the population.

Our subgroup analysis results revealed a significant difference in the prevalence of compassion fatigue among different categories of healthcare professionals. As expected, nurses reported the highest rates of compassion fatigue at 80%, followed by physicians at 59%, and lastly nursing students at 50%. While our results, along with numerous other studies [3], have reported a high prevalence of compassion fatigue among healthcare professionals, nurses are more affected. The high prevalence of compassion fatigue among nurses is evident in the present study. Due to the nature of their work, nurses are more psychologically and physically involved in patient care, often working longer hours than physicians, which could explain these results. Nurses provide the majority of healthcare services and have the most direct contact with patients’ traumatic experiences. Additionally, it is important to note that there are numerous confounding factors to consider, including gender vulnerability and mean age. However, elevated levels of compassion fatigue among nurses had already been identified prior to the pandemic. For example, Lu and colleagues reported rates of 81.8%, and Wang and colleagues reported rates of 81.6% [33, 34].

Our results showed that healthcare professionals during the COVID-19 pandemic were at a higher risk of experiencing compassion fatigue compared to their counterparts in the pre-COVID-19 era. The overall prevalence of compassion fatigue before COVID-19 was found to be 66% (95% CI: 41.91%, 12 = 27%) among healthcare professionals. During COVID-19, the prevalence of compassion fatigue was shown to be 74% (95% CI: 63.85%, 12 = 88.73%) among healthcare professionals. The pandemic’s escalation in cases, workload, deaths, and exposure to other traumatic events could explain the increase in compassion fatigue among healthcare professionals [4]. Our findings are consistent with a review by Xie and colleagues among emergency nurses conducted prior to the COVID-19 pandemic [32]. Conversely, studies conducted before the COVID-19 pandemic reported mild cases of compassion fatigue among healthcare professionals [35]. Thus, our results indicate that the pandemic and its impact on healthcare professionals surpassed the levels reported in the pre-COVID-19 era. COVID-19 exposed healthcare professionals to traumatic events such as patient deaths and suffering, resulting in the development of compassion fatigue [30]. Conversely, differences in the prevalence of compassion fatigue could be attributed to several confounding factors, including variations in populations, sample sizes, and regions between pre- and post-COVID-19 pandemic studies.

Our sub-group analysis revealed a significant geographical variation in the prevalence of compassion fatigue. Of the two regions in Sub-Saharan Africa where we found studies on compassion fatigue, Eastern Africa had a higher overall prevalence (74%) compared to 64% in southern Africa. This difference can be attributed to the publication of more studies on compassion fatigue in southern Africa compared to eastern Africa. Eastern Africa reports a scarcity of resources, especially psychological resources aimed at alleviating compassion fatigue, compared to southern Africa [36]. Studies comparing regions have reported differences in compassion fatigue between high- and low-income countries [37]. These studies have shown that low-income countries tend to report higher levels of compassion fatigue among healthcare professionals than high-income countries [37]. Other differences in the prevalence of compassion fatigue among healthcare professionals can be attributed to variations in healthcare systems.

In addressing compassion fatigue among healthcare workers in Sub-Saharan Africa, several practical strategies can be recommended. Firstly, implementing regular self-care routines such as brief mindfulness exercises or stretching during shifts can provide immediate relief. Peer support groups where colleagues can share experiences and coping mechanisms can be established, fostering a sense of camaraderie and understanding. Providing access to on-site counseling services or virtual mental health resources tailored to the cultural context can offer timely support. Additionally, organizing rotating schedules to ensure sufficient breaks and rest periods, combined with leadership encouragement of these practices, can significantly alleviate the strain. Finally, incorporating training sessions on recognizing compassion fatigue signs and effective stress management techniques can empower healthcare workers to proactively address their mental well-being.

### Limitations of the study

The meta-analysis results unveiled a considerable degree of heterogeneity, an expected outcome due to the varied locations and timings of the studies conducted both before and during the COVID-19 pandemic. Variations in sample sizes across the included studies also likely contributed to this heterogeneity. Notably, there is a scarcity of studies examining compassion fatigue among healthcare professionals in Sub-Saharan Africa, with no identified studies from West African countries. The inclusion of gray literature could potentially impact the robustness of our findings, and the decision to limit our synthesis to quantitative studies may have resulted in overlooking pertinent qualitative research. Future studies could examine into these qualitative findings. Moreover, the reliance on self-reported questionnaires in uncontrolled settings introduces the possibility of reporting bias. Also, the limited number of studies comparing compassion fatigue between pre- and during the COVID-19 era at the time of this review restricts the depth of discussion on these subgroup results. Lastly, our study deviated from the PROSPERO protocol, which specifies the year 2000 as the start date for primary study searches, which could potentially lead to the exclusion of relevant bibliographic material and introduce publication bias.

## Conclusion

Our results indicate that almost 3 in 4 healthcare professionals in Sub-Saharan Africa experience compassion fatigue. The results also show that compassion fatigue increased as a result of COVID-19. The high prevalence underscores the importance of addressing and mitigating compassion fatigue in Sub-Saharan Africa to support the mental health and emotional wellbeing of healthcare professionals who dedicate themselves to helping others in challenging circumstances.

## Supporting information

S1 Checklist PRISMA 2009 checklist. (DOC)

S1 Text Search strategy. (DOCX)

S1 Table Risk of bias assessment. (DOCX)

S1 Data Data. (XLSX)
