# Supplementary material for: A systematic review and meta-analysis of compassion fatigue among healthcare professionals before and during COVID-19 in Sub-Saharan Africa
Source: PLOS Glob Public Health. 2024 Jun 21;4(6):e0003388. doi: 10.1371/journal.pgph.0003388 (PMC11192372; doi:10.1371/journal.pgph.0003388)
Supplement: S1 Text — (DOCX) [file pgph.0003388.s002.docx]

**Search strategy in PUBMED**

| **Level** | **String** | **Number** |
| --- | --- | --- |
| #1 | “Compassion fatigue” OR “Cost of care” OR “secondary traumatic stress” OR “vicarious traumatization” OR “secondary survivor” OR “secondary victimization” | 5768 |
| #2 | “Healthcare worker” OR Physician OR “medical doctor” OR specialist OR “general practitioner” OR “general practice physician” OR “health staff” OR “health personnel” OR “healthcare provider” OR “healthcare professionals” OR psychiatrist | 1969317 |
| #3 | Covid-19 OR COVID 19 OR COVID-19 OR “COVID-19 virus disease” OR “COVID-19 infection” OR “COVID-19 virus infection” OR 2019-nCoV OR “2019-nCoV disease” OR “corona virus disease” OR “novel corona virus infection” OR “SARS corona virus 2 Infection” OR “SARS CoV 2 infection” OR “COVID-19 pandemic” | 587210 |
| #4 | “Sub-Saharan Africa” OR Africa OR “Africa south of the Sahara” OR Angola OR Benin OR Botswana OR “Burkina Faso” OR Burundi OR Cameroon OR “Cape Verde” OR “Central African Republic” OR Chad OR Comoros OR “Republic of the Congo” OR “Democratic Republic of the Congo” OR “Cote d'Ivoire” OR Djibouti OR “Equatorial Guinea” OR Eritrea OR Ethiopia OR Gabon OR Gambia OR Ghana OR Guinea OR Guinea-Bissau OR Kenya OR Liberia OR Madagascar OR Malawi OR Mali OR Mauritania OR Mauritius OR Mozambique OR Namibia OR Niger OR Nigeria OR Rwanda OR “Sao Tome and Principe” OR Senegal OR Seychelles OR “Sierra Leone” OR Somalia OR “South Africa” OR “South Sudan” OR Sudan OR Swaziland OR Tanzania OR Togo OR Uganda OR Zambia OR Zimbabwe | 1001176 |
| #5 | #1 AND #2 AND #3 AND 4 | 538 |
| #6 | #5 AND Date (January 1, 2010 to July 31, 2023) | 534 |

**Search strategy in Google scholar**

| #1 | With all of the words | Country |
| --- | --- | --- |
| #2 | With the exact phrase | “compassion fatigue” |
| #3 | With at least one of the words | "Secondary traumatic stress" "vicarious traumatization" "healthcare workers" "health professionals" nurses "medical doctors" "health workers" |
| #4 | Anywhere in the article | #1 AND #2 AND #3 Limit to return articles dated between 2010 and 2023 |

**Search strategy in ScienceDirect**

| Level | String |
| --- | --- |
| #1 | “Predictors” OR “Associated factors” OR “Determinants” |
| #2 | “compassion fatigue” OR "Secondary traumatic stress" OR "vicarious traumatization" |
| #3 | "Health workers" OR "Healthcare workers" |
| #4 | Africa |
| #5 | #1 AND #2 AND #3 AND #4 Limit by time span 2020-2022 |
