# Supplementary material for: A systematic review and meta-analysis of compassion fatigue among healthcare professionals before and during COVID-19 in Sub-Saharan Africa
Source: PLOS Glob Public Health. 2024 Jun 21;4(6):e0003388. doi: 10.1371/journal.pgph.0003388 (PMC11192372; doi:10.1371/journal.pgph.0003388)
Supplement: S1 Table — (DOCX) [file pgph.0003388.s003.docx]

**Table: Quality of included studies**

| **Study** | **Representativeness** | **Sample size** | **Response rate** | **Screening tool** | **Compatibility** | **Outcome assessment** | **Statistical test** | **Total score** | **Quality** |
| --- | --- | --- | --- | --- | --- | --- | --- | --- | --- |
| Donald | 1 | 0 | 1 | 2 | 0 | 2 | 1 | 7 | Good |
| Mohammed | 1 | 1 | 1 | 2 | 1 | 2 | 1 | 9 | Good |
| Amir | 1 | 1 | 1 | 2 | 1 | 2 | 1 | 9 | Good |
| Amir | 1 | 0 | 1 | 2 | 1 | 2 | 1 | 8 | Good |
| Teresa | 0 | 0 | 1 | 2 | 1 | 2 | 1 | 7 | Good |
| Mathias | 1 | 0 | 1 | 2 | 0 | 2 | 0 | 6 | Good |
| Phindile | 1 | 0 | 0 | 2 | 0 | 2 | 1 | 6 | Good |
| Beatrice | 0 | 0 | 0 | 2 | 1 | 2 | 1 | 6 | Good |
| Almaz | 0 | 0 | 0 | 0 | 1 | 0 | 1 | 2 | Poor |
| Wentzel | 0 | 0 | 0 | 2 | 0 | 2 | 1 | 5 | Poor |
| Addisu | 1 | 0 | 1 | 2 | 1 | 2 | 1 | 8 | Good |
